# The evaluation of cytokine profiles in atopic dermatitis patients treated with dupilumab: association with ocular complications

**DOI:** 10.3389/fimmu.2026.1873001

**Published:** 2026-08-06

**Authors:** Jarmila Čelakovská, Eva Čermáková, Petra Boudkova, Ctirad Andrýs

**Affiliations:** 1 Department of Dermatology and Venereology Faculty Hospital and Medical Faculty of Charles University, Hradec Králové, Czechia; 2 Department of Medical Biophysics, Medical Faculty of Charles University, Hradec Králové, Czechia; 3 Department of Clinical Immunology and Allergy, Faculty Hospital and Medical Faculty of Charles University, Hradec Králové, Czechia

**Keywords:** atopic dermatitis, blepharitis, conjunctivitis, cytokines, dupilumab, dupilumab complications, keratitis, ocular inflammation

## Abstract

**Background:**

Dupilumab is an effective treatment for moderate-to-severe atopic dermatitis (AD), yet a substantial proportion of patients develop ocular complications collectively termed dupilumab-associated ocular surface disease (DAOSD). Despite multiple proposed mechanisms - including goblet cell depletion, mucin deficiency, Th1/Th17 immune shifts, and microbiome alterations - no upstream epithelial cytokine marker has been clearly identified.

**Objective:**

To evaluate whether plasma cytokines differ between AD patients treated with dupilumab who develop ocular complications and those without ocular involvement.

**Methods:**

Complex dermatological examination was performed in adult AD patients receiving dupilumab for ≥24 months. Plasma cytokines (IFN-γ, TNF-α, IL−23, IL−2, IL−31, IL−6, IL−17A, IL−17E, IL−12p70, TSLP, IL−4, IL−5, IL−13, IL−10, IL−33) were quantified under unstimulated and PMA/ionomycin−stimulated conditions in winter and summer seasons. Differences between groups were assessed using non-parametric tests (Mann–Whitney U test and Kolmogorov–Smirnov test). To account for multiple comparisons across the large number of tested cytokines, false discovery rate (FDR) correction was performed using the Benjamini–Hochberg procedure with a significance threshold of q = 0.05. Receiver operating characteristic (ROC) analysis was performed to evaluate biomarker potential to explore potential discriminative performance. AUC values were calculated using empirical (non-parametric) ROC analysis.

**Results:**

During the summer period, stimulated thymic stromal lymphopoietin (TSLP) levels were nominally higher in patients with ocular complications compared with those without (p = 0.045). However, this difference did not remain statistically significant after FDR correction. In winter, stimulated TSLP showed a non-significant trend toward higher levels (p = 0.088). No other cytokines differed between groups. ROC analysis demonstrated moderate discriminative ability of stimulated TSLP (AUC 0.81 in summer; 0.70 in winter), although confidence intervals were wide.

**Conclusion:**

Stimulated TSLP may represent a preliminary observation requiring confirmation in dupilumab-treated AD patients with ocular complications.

## Introduction

Dupilumab, a monoclonal antibody targeting the IL-4 receptor alpha subunit, has markedly improved outcomes in patients with moderate-to-severe atopic dermatitis (AD). Nevertheless, ocular complications - including conjunctivitis, keratitis, and blepharitis - remain a frequent adverse effect (1–7). Current literature highlights several pathophysiological mechanisms, most notably conjunctival goblet-cell depletion, mucin deficiency, alterations in the ocular microbiome, and changes in T-helper cell pathways (1–5). Despite these insights, cytokine profiling has not yet identified upstream biomarkers that predict susceptibility to dupilumab-associated ocular surface disease (DAOSD). The most consistently documented mechanism involves a marked reduction of conjunctival goblet cells following IL-4/IL-13 blockade (1–5). A comprehensive review published in 2025 further supports this concept, emphasizing goblet-cell loss, mucin imbalance, immune dysregulation, and *Demodex*-associated inflammation as central drivers of DAOSD (6). Another major contributing factor appears to be immune shifts caused by IL-4/IL-13 inhibition. While dupilumab suppresses Th2-driven inflammation effectively, it may promote compensatory activation of Th1 and Th17 pathways (7–9). These shifts have been proposed to contribute to blepharitis, keratitis, and meibomian-gland dysfunction, and recent analyses describe DAOSD as partially rooted in Th1/Th17-mediated inflammation. Importantly, such immune alterations occur downstream of epithelial cytokines (7–9).

Our study builds on our previous work evaluating cytokine patterns in AD patients without systemic therapy, those treated with dupilumab, and healthy controls (10). In that earlier analysis, we demonstrated that although dupilumab effectively suppresses Th2 signaling, it does not fully normalize immune balance: residual Th17 activity and innate inflammatory responses persist. Elevated TSLP in AD patients with and without dupilumab therapy compared to healthy controls and IL-6 suggested ongoing epithelial stress and innate immune activation, while reduced IL-23 and IL-2 under stimulation indicated impaired adaptive immune responsiveness in AD patients (10).

### Objective

The aim of the present study is to determine whether plasma cytokines (IFN-γ, TNF-α, IL-23, IL-2, IL-31, IL-6, IL-17A, IL-17E, IL-12p70, TSLP, IL-4, IL-5, IL-13, IL-10, and IL-33) differ between AD patients receiving dupilumab who develop ocular complications and those who do not. The selected cytokines were chosen to reflect key immune pathways relevant to AD and dupilumab therapy, including Th2 (IL-4, IL-5, IL-13), Th1 (IFN-γ), Th17 (IL-17A, IL-23), regulatory cytokines (IL-10), innate inflammatory markers (IL-6, TNF-α), and epithelial-derived alarmins (TSLP, IL-33). In particular, TSLP was included due to its central role in epithelial - immune crosstalk and its potential involvement in ocular surface inflammation.

## Methods

We included adult patients with AD treated with dupilumab for at least 24 months. All patients were recruited at a single center in the Czech Republic, and the cohort was ethnically homogeneous (predominantly Central European origin). Based on data from the nearest phenological station Běleč nad Orlicí (241 m above sea level), 50°12′ N, 15°56′ E), which is located in the immediate vicinity of Hradec Králové (East Bohemia region in Czech Republic), the following species are present from important pollen allergens: owing to the sandy substrate, white birch (Betula pendula) and wood pine (Pinus sylvestris) predominate, as well as common hazel (Corylus avellana). There is also abundant sticky alder (Alnus glutinosa), willow (Salix caprea), hornbeam (Carpinus betulus), summer oak (Quercus robur), spruce (Picea excelssa), deciduous larch (Larix decidua), Heartworm (Tilia cordata). For trees flowering in the phenological pre-spring (hazel and alder), the shift is 11 to 29 days. The most important pollen allergen, white birch blooms on average more than 10 days earlier. Birch and other related trees of the families Betulaceae and Fagaceae (alder, hazel, oak, hornbeam, chestnut, and beech) constitute the birch homologous group.

Patients were categorized according to the presence or absence of ocular complications after starting dupilumab therapy. Ocular complications included conjunctivitis, blepharitis, and keratitis diagnosed by ophthalmologic examination. However, standardized grading systems, severity scores, and tear film assessments were not uniformly available.

The group with ocular complications (conjunctivitis, blepharitis, and keratitis) included patients who developed new ocular symptoms after initiating dupilumab therapy. Patients in the ocular complication group were further characterized according to the clinical status of ocular disease at the time of blood sampling (active vs inactive). Active disease was defined by the presence of ongoing ocular symptoms requiring treatment, whereas inactive disease referred to patients with a previous history of dupilumab-associated ocular complications that had resolved prior to sampling.

The group without ocular complications consisted of patients who did not develop any new ocular issues after starting dupilumab treatment. The complex dermatological examination was performed in adult AD patients receiving dupilumab for ≥24 months. AD severity was assessed using the Eczema Area and Severity Index (EASI) and the Scoring Atopic Dermatitis (SCORAD) system. Additionally, patients completed the Patient-Oriented Eczema Measure (POEM) and the Dermatology Life Quality Index (DLQI) to evaluate subjective symptoms and quality of life.

### Laboratory examination

Whole blood samples were stimulated with phorbol 12-myristate 13-acetate (PMA, 25 ng/mL) and ionomycin (1 µg/mL) and incubated for 4 hours at 37 °C. This protocol induces non-specific activation of T cells and innate immune cells, allowing assessment of maximal cytokine production capacity. Unstimulated samples were processed in parallel.

This approach reveals the functional capacity of immune cells to produce cytokines upon activation, allowing detection of latent immune responses that may not be evident in baseline plasma. Cytokine concentrations were measured using the Human Cytokine Luminex Assay “Human Luminex Discovery Assay (catalog number LXSAHM-05). Cytokine testing was performed in pollen season and outside the pollen season.

### Statistic

We evaluated whether **p**lasma cytokines levels (IFN-γ, TNF-α, IL−23, IL−2, IL−31, IL−6, IL−17A, IL−17E, IL−12p70, TSLP, IL−4, IL−5, IL−13, IL−10, IL−33) differ in patients without and with ocular complications. Differences between groups were assessed using non-parametric tests (Mann–Whitney U test and Kolmogorov–Smirnov test).

To account for multiple comparisons across the large number of tested cytokines, false discovery rate (FDR) correction was performed using the Benjamini–Hochberg procedure with a significance threshold of q = 0.05. The correction was applied across all cytokine comparisons within each season separately, including both unstimulated and stimulated conditions. Adjusted p-values (q-values) were calculated using the Benjamini–Hochberg procedure and are reported alongside raw p-values. The adjusted p-values represent the false discovery rate (FDR)-corrected significance levels. FDR correction was performed separately for each season because cytokine measurements were analyzed as independent seasonal datasets with potentially distinct environmental influences.

Receiver operating characteristic (ROC) analysis was performed to evaluate biomarker potential to explore potential discriminative performance. We show the detailed statistical analysis. AUC values were calculated using empirical (non-parametric) ROC analysis.

## Results

We included 26 adults with AD (10 women, 16 men) who had been treated with dupilumab for at least 24 months. Patients were categorized according to the presence (n = 11) or absence (n = 15) of chronic ocular complications. The group with ocular complications included patients who developed new ocular symptoms after initiating dupilumab therapy. The group without ocular complications consisted of patients who did not develop any new ocular issues after starting dupilumab treatment. For the summer dataset included a subset of patients (n = 19) due to missing data for stimulated TSLP measurements in some samples.

**Table 1** summarizes the patient characteristics. We did not observe differences in AD severity (according to SCORAD and EASI). However, patients with ocular complications reported higher impairment in quality-of-life assessments. Polyvalent allergy to molecular allergens was demonstrated in both groups.

**Table 1:** Participant characteristics: Means (ranges) are shown for SCORAD, EASI, POEM, DLQI.

	Eye complications - conjunctivitis, blepharitis, and keratitis	
	Eye complications yes 11 patients (9 men, 2 women) active disease in 11 patients, inactive disease 0 patients	Eye complications no 15 patients (7 men, 8 women)
Age of patients	Mean age of 41.2 years (32.5- 48.3)	Mean age of 40.1 years (35.2-68.2)
SCORAD	10.2	9.8
EASI	9.1	9.0
POEM	4.1 (2 –6)	3.9 (2 –6)
DLQI	5.2	2.1
Asthma bronchiale	7 (63.6%)	9 (60%)

Explanation.

Active disease: ongoing symptoms requiring treatment. All patients with ocular complications had active disease at the time of sampling.

Inactive disease: previous complications resolved before sampling in none of patients.

Disease severity and patient-reported burden were assessed using validated clinical and quality-of-life instruments. Atopic dermatitis severity was evaluated with the Scoring Atopic Dermatitis (SCORAD) index and the Eczema Area and Severity Index (EASI). SCORAD values <25 indicated mild, 25–50 moderate, and >50 severe disease, while EASI scores were interpreted as mild (≤7), moderate (7–21), or severe (>21) eczema. SCORAD integrates objective clinical findings with patient-reported symptoms, whereas EASI provides a purely objective physician-rated assessment of inflammatory skin involvement. Health-related quality of life was measured using the Dermatology Life Quality Index (DLQI), with scores of 0–1 indicating no effect, 2–5 small, 6–10 moderate, 11–20 very large, and 21–30 extremely large impact on quality of life. Patient-reported disease symptoms were further assessed using the Patient-Oriented Eczema Measure (POEM), classified as clear/almost clear (0–2), mild (3–7), moderate (8–16), severe (17–24), or very severe (25–28).

We show the occurrence of bronchial asthma, allergic rhinitis in patients with and without eye complications.

The plasma cytokines levels (IFN-γ, TNF-α, IL−23, IL−2, IL−31, IL−6, IL−17A, IL−17E, IL−12p70, TSLP, IL−4, IL−5, IL−13, IL−10, IL−33) in patients without and with ocular complications in summer season are recorded in **Table 2**. Means are shown for completeness; statistical interpretation based on medians. During the summer season, stimulated TSLP levels nominally higher in patients with ocular complications compared with those without ocular involvement (*p = 0.0446*); this observed difference in stimulated TSLP should be interpreted cautiously given the multiple comparisons performed and the limited sample size. Due to the non-normal (skewed) distribution of the data, results are presented as median (interquartile range, 25th–75th percentile) for patients with and without eye complications, separately for summer seasons. Values are presented in pg/mL under unstimulated (n) and stimulated (s) conditions (**Supplementary Table to Table 2**).

**Table 2:** Plasma cytokine levels in patients with and without ocular complications during the summer season.

Cytokine	With ocular complications (mean ± SD)	Eye complications no (mean ± SD)	P value
IL-4 (un)	0.10 ± 0.20	0.003 ± 0.010	0.904
IL-4 (s)	0.10 ± 0.18	0.005 ± 0.016	0.230
IL-13 (un)	0.14 ± 0.31	0.00 ± 0.00	NA
IL-13 (s)	0.14 ± 0.30	0.21 ± 0.52	0.833
IL-5 (un)	0.00 ± 0.00	0.00 ± 0.00	NA
IL-5 (s)	0.016 ± 0.025	0.035 ± 0.038	0.220
IL-10 (un)	0.00 ± 0.00	0.00 ± 0.00	NA
IL-10 (s)	0.29 ± 0.31	0.36 ± 0.25	0.348
IL-33 (un)	0.18 ± 0.53	0.00 ± 0.00	NA
IL-33 (s)	0.15 ± 0.44	0.00 ± 0.00	NA
IL-6 (un)	4.06 ± 9.60	1.94 ± 2.16	0.787
IL-6 (s)	123.03 ± 140.21	120.43 ± 68.00	0.575
IL-17A (un)	3.73 ± 8.12	0.00 ± 0.00	NA
IL-17A (s)	492.05 ± 383.24	296.49 ± 137.76	0.442
IL-17E/IL-25 (un)	0.13 ± 0.40	0.00 ± 0.00	NA
IL-17E/IL-25 (s)	39.04 ± 86.15	0.00 ± 0.00	NA
IL-12p70 (un)	405.83 ± 668.57	36.87 ± 73.02	0.060
IL-12p70 (s)	432.56 ± 631.89	41.23 ± 84.75	0.060
TSLP (un)	15.54 ± 29.28	1.52 ± 3.30	0.200
TSLP (s)	15.20 ± 24.29	1.95 ± 3.69	**0.045**
IL-23 (un)	519.01 ± 841.74	29.67 ± 80.52	0.252
IL-23 (s)	2209.17 ± 952.41	1666.02 ± 532.64	0.111
IL-2 (un)	0.69 ± 1.80	0.00 ± 0.00	NA
IL-2 (s)	11492.84 ± 6112.74	10443.11 ± 4738.57	0.967
IL-31 (un)	692.95 ± 1503.74	86.55 ± 188.81	0.442
IL-31 (s)	658.94 ± 1335.93	132.65 ± 156.08	0.442
IFN-γ (un)	1.63 ± 2.03	0.89 ± 1.95	0.340
IFN-γ (s)	6370.10 ± 1354.22	6717.43 ± 1186.18	0.653
TNF-α (un)	8.40 ± 11.84	1.95 ± 2.28	0.539
TNF-α (s)	4192.33 ± 2705.36	3346.90 ± 1150.81	0.575

Bold p values indicate statistical significance (p < 0.05).

NA: p value not calculated where one or both groups were zero.

Values are presented as mean ± SD (pg/mL) under unstimulated (un) and stimulated (s) conditions. Group comparisons were performed using non-parametric tests.

The plasma cytokines levels (IFN-γ, TNF-α, IL−23, IL−2, IL−31, IL−6, IL−17A, IL−17E, IL−12p70, TSLP, IL−4, IL−5, IL−13, IL−10, IL−33) in patients without and with ocular complications in winter season are recorded in **Table 3**. Means are shown for completeness; statistical interpretation based on medians. In winter, stimulated TSLP levels showed a borderline increase (p = 0.0885). No significant differences were observed in any other cytokines across seasons or stimulation conditions. Due to the non-normal (skewed) distribution of the data, results are presented as median (interquartile range, 25th–75th percentile) for patients with and without eye complications, separately for summer seasons. Values are presented in pg/mL under unstimulated (n) and stimulated (s) conditions (**Supplementary Table to Table 3**).

**Table 3:** Plasma cytokine levels in patients with and without ocular complications during the winter season.

Cytokine	With ocular complications (mean ± SD)	Eye complications no (mean ± SD)	P value
IL-4 (un)	0.08 ± 0.18	0.02 ± 0.08	1.000
IL-4 (s)	0.08 ± 0.17	0.10 ± 0.12	0.259
IL-13 (un)	0.10 ± 0.28	0.02 ± 0.08	1.000
IL-13 (s)	0.09 ± 0.26	0.16 ± 0.37	0.598
IL-5 (un)	0.00 ± 0.00	0.00 ± 0.00	NA
IL-5 (s)	0.07 ± 0.11	0.09 ± 0.10	0.405
IL-10 (un)	0.00 ± 0.00	0.00 ± 0.00	NA
IL-10 (s)	0.47 ± 0.47	0.51 ± 0.45	0.678
IL-33 (un)	0.10 ± 0.35	0.12 ± 0.45	0.911
IL-33 (s)	0.09 ± 0.29	0.12 ± 0.45	0.911
IL-6 (un)	8.65 ± 18.21	2.39 ± 3.06	0.621
IL-6 (s)	184.26 ± 95.29	204.11 ± 124.26	0.917
IL-17A (un)	26.41 ± 51.02	2.56 ± 7.69	0.229
IL-17A (s)	450.11 ± 225.74	378.29 ± 229.32	0.436
IL-17E/IL-25 (un)	12.68 ± 42.06	7.33 ± 26.91	0.852
IL-17E/IL-25 (s)	20.28 ± 48.70	7.33 ± 26.91	1.000
IL-12p70 (un)	452.77 ± 880.49	155.03 ± 373.85	0.529
IL-12p70 (s)	469.41 ± 826.95	158.29 ± 355.16	0.253
TSLP (un)	13.45 ± 27.20	6.48 ± 17.52	0.119
TSLP (s)	13.32 ± 23.24	7.46 ± 19.10	0.088
IL-23 (un)	197.59 ± 397.03	178.64 ± 385.30	0.515
IL-23 (s)	2070.66 ± 507.69	2064.36 ± 452.55	0.959
IL-2 (un)	0.94 ± 3.10	0.00 ± 0.00	NA
IL-2 (s)	13852.88 ± 3762.72	19733.45 ± 11998.33	0.188
IL-31 (un)	252.99 ± 409.97	106.49 ± 290.59	0.166
IL-31 (s)	271.12 ± 367.42	229.94 ± 392.21	0.223
IFN-γ (un)	3.68 ± 8.78	0.31 ± 0.49	0.104
IFN-γ (s)	7385.06 ± 831.54	7790.69 ± 932.48	0.299
TNF-α (un)	6.20 ± 8.28	5.07 ± 6.93	0.833

NA: p value not calculated where one or both groups were zero.

Values are presented as mean ± SD (pg/mL) under unstimulated (un) and stimulated (s) conditions.

After application of the Benjamini -Hochberg correction, none of the observed differences remained statistically significant in either the summer or winter datasets. Adjusted p-values for all comparisons are presented in **Table 4a** for summer season and **Table 4b** for winter season.

**Table 4a:** We performed Benjamini-Hochberg FDR correction (FDR = 0.05) for summer season.

Cytokine	P value	Rank	Adjusted p value (q value)
TSLP (s)	0.045	1	0.420
IL-12p70 (un)	0.06	3	0.420
IL-12p70 (s)	0.06	3	0.420
IL-23 (s)	0.111	4	0.583
TSLP (un)	0.2	5	0.662
IL-5 (s)	0.22	6	0.662
IL-4 (s)	0.23	7	0.662
IL-23 (un)	0.252	8	0.662
IFN-γ (un)	0.34	9	0.714
IL-10 (s)	0.348	10	0.714
IL-17A (s)	0.442	13	0.714
IL-31 (un)	0.442	13	0.714
IL-31 (s)	0.442	13	0.714
TNF-α (un)	0.539	14	0.755
IL-6 (s)	0.575	16	0.755
TNF-α (s)	0.575	16	0.755
IFN-γ (s)	0.653	17	0.807
IL-6 (un)	0.787	18	0.918
IL-13 (s)	0.833	19	0.921
IL-4 (un)	0.904	20	0.949
IL-2 (s)	0.967	21	0.967

We excluded from the calculations those quantities where the comparison was not performed (p-value N/A). Cytokines are ranked by p-value. P-value adjusted are presented. The calculations show that after the correction, no comparison is significant.

The Benjamin-Hochberg FDR correction - the calculations in Excel is performed according to https://www.r-bloggers.com/2023/07/the-benjamini-hochberg-procedure-fdr-and-p-value-adjusted-explained/.

**Table 4b:** We performed Benjamini-Hochberg FDR correction (FDR = 0.05) for winter season.

Cytokine	P value	Rank	Adjusted p value (q value)
TSLP (s)	0.088	1	0.748
IFN-γ (un)	0.104	2	0.748
TSLP (un)	0.119	3	0.748
IL-31 (un)	0.166	4	0.748
IL-2 (s)	0.188	5	0.748
IL-31 (s)	0.223	6	0.748
IL-17A (un)	0.229	7	0.748
IL-12p70 (s)	0.253	8	0.748
IL-4 (s)	0.259	9	0.748
IFN-γ (s)	0.299	10	0.777
IL-5 (s)	0.405	11	0.945
IL-17A (s)	0.436	12	0.945
IL-23 (un)	0.515	13	0.982
IL-12p70 (un)	0.529	14	0.982
IL-13 (s)	0.598	15	1
IL-6 (un)	0.621	16	1
IL-10 (s)	0.678	17	1
TNF-α (un)	0.833	18	1
IL-17E/IL-25 (un)	0.852	19	1
IL-33 (un)	0.911	21	1
IL-33 (s)	0.911	21	1
IL-6 (s)	0.917	22	1
IL-23 (s)	0.959	23	1
IL-4 (un)	1	26	1
IL-13 (un)	1	26	1
IL-17E/IL-25 (s)	1	26	1

We excluded from the calculations those quantities where the comparison was not performed (p-value N/A). Cytokines are ranked by p-value. P-value adjusted are presented. The calculations show that after the correction. no comparison is significant. The Benjamin-Hochberg FDR correction - the calculations in Excel is performed according to https://www.r-bloggers.com/2023/07/the-benjamini-hochberg-procedure-fdr-and-p-value-adjusted-explained/.

Receiver operating characteristic (ROC) analysis was performed for stimulated TSLP for summer and winter. The area under the curve (AUC) was 0.81 in summer and 0.70 in winter, indicating moderate discriminative ability (**Table 5**).

**Table 5:** Receiver operating characteristic (ROC) analysis was performed for stimulated TSLP for summer and winter season.

Parameter	Summer	Winter
AUC (95% CI)	0.81 (0.47–0.94)	0.70 (0.42-0.86)
p-value	0.0025	0.0353
Cutoff (TSLP_s)	≥1.21 pg/mL	≥3.9 pg/mL (exploratory cutoff)
Sensitivity	88.9%	~63.6%
Specificity	70.0%	~80%

The area under the curve (AUC) was 0.81 in summer and 0.70 in winter. AUC values were calculated using empirical (non-parametric) ROC analysis.

### Summer ROC

ROC analysis of stimulated TSLP demonstrated moderate discriminative ability for ocular complications, with an AUC of 0.81 (95% CI 0.47 -0.94; p = 0.0025). The optimal cutoff value (≥1.21 pg/mL), determined by the Youden index, yielded a sensitivity of 88.9% and specificity of 70.0% (accuracy 78.9%), (**Table 5**, **Supplementary Table 5** - summer season). The detailed analysis is recorded in Complement to **Table 5** for summer season. For the summer dataset included a subset of patients (n = 19) due to missing data for stimulated TSLP measurements in some samples.

### Winter ROC

In the winter dataset, discriminative ability was lower - AUC of 0.70 ((95% CI 0.42-0.86; p = 0.0353) with reduced sensitivity and specificity, indicating less robust performance, **Table 5**, **Supplementary Table to Table 5** - winter season. The detailed analysis is recorded in Complement to **Table 5** for winter season.

Because TSLP was identified from the same dataset and did not remain significant after FDR correction, the ROC results should be interpreted as exploratory only and do not support clinical diagnostic use.

## Discussion

In this study, we evaluated systemic cytokine profiles in dupilumab-treated patients with atopic dermatitis in relation to the development of ocular complications. The principal finding of this study is a nominal increase in stimulated TSLP levels in patients with ocular complications during the summer season. However, this difference did not remain statistically significant after correction for multiple testing and should therefore be interpreted with caution. These findings support only a hypothesis-generating observation rather than evidence of a definitive biomarker. While TSLP is known to play a role in epithelial–immune interactions, the present data reflect systemic cytokine responses under stimulation and do not allow conclusions regarding the cellular source or ocular surface origin of TSLP.

In our study, all patients in the ocular complication group had active ocular disease at the time of sampling; therefore, the observed differences may reflect concurrent inflammation rather than susceptibility to develop ocular complications. On the other hand, ocular complications occurred after initiation of dupilumab and have persisted continuously to varying degrees since then.

Receiver operating characteristic (ROC) analysis was performed as an exploratory assessment of the potential discriminative performance of stimulated TSLP. In the summer dataset, the area under the curve (AUC) was 0.81 (95% CI 0.47–0.94; p = 0.0025), whereas in winter the AUC was 0.70 with wide confidence intervals (95% CI 0.42-0.86; p = 0.0353), indicating limited and uncertain discriminative ability. Due to the small sample size, absence of validation, and lack of statistical significance after FDR correction, these findings should be interpreted strictly as exploratory. The reported cutoff values, sensitivity, and specificity represent non-validated estimates and do not support diagnostic or predictive use.

Importantly, no other cytokines, including those representing Th1, Th2, Th17, or innate immune pathways, differentiated patients with and without ocular complications. This lack of systemic differences suggests that ocular complications are not primarily driven by generalized immune dysregulation, but may instead reflect localized epithelial or ocular surface mechanisms.

While TSLP is commonly described as an epithelial-derived alarmin, the use of whole-blood stimulation in this study does not allow identification of the cellular source of cytokine production. Therefore, the results should be interpreted as reflecting systemic immune activation rather than direct evidence of ocular surface or epithelial mechanisms.

The observed seasonal pattern, with a stronger signal in summer, further supports this interpretation. Environmental factors such as allergen exposure, UV radiation, and airborne irritants may modulate epithelial cytokine responses. As TSLP is known to be inducible by epithelial stress, this seasonal variability is biologically plausible, although it requires further confirmation.

Histopathological findings provide additional context. Patients who develop conjunctivitis during dupilumab therapy demonstrate a profound reduction in conjunctival goblet cells (3.3 cells/mm vs. 32.3 cells/mm in controls), accompanied by infiltration of CD3+/CD4+ T cells and eosinophils (6). This goblet cell loss leads to mucin deficiency, tear film instability, and subsequent ocular surface inflammation, and is considered a key mechanism underlying DAOSD (6). These local changes support the concept that epithelial barrier dysfunction plays a central role in disease pathogenesis.

Our results also build on findings from our previous study (10). Under unstimulated conditions, we previously observed elevated TSLP in both groups of AD patients, indicating persistent epithelial barrier stress (10). At the same time, increased IL-17A and TNF-α suggested residual pro-inflammatory and Th17 activity despite Th2 blockade, while reduced IFN-γ was consistent with Th2 dominance. Under stimulated conditions, we observed lower IL-23 and IL-2, indicating impaired adaptive immune responsiveness, alongside elevated IL-6 reflecting ongoing innate inflammatory activation (10).

In the present study, TSLP differentiated patient groups only under stimulated conditions, not at baseline. This distinction is important, as stimulated cytokine assays reflect functional immune capacity rather than resting levels. Dupilumab effectively suppresses Th2 pathways, but its influence on epithelial-derived cytokines such as TSLP may become apparent only after immune activation. Elevated stimulated TSLP levels therefore suggest that, in patients who develop ocular complications, immune cells retain an increased capacity to produce TSLP upon stimulation. This finding may reflect a latent or inducible epithelial stress response that is not detectable under unstimulated conditions, indicating a primed epithelial–immune axis.

The stronger association observed during the summer period further supports the role of environmental modulation. Increased exposure to pollens, UV radiation, wind, and airborne irritants can aggravate epithelial surfaces (11–14). As TSLP is rapidly released by epithelial cells in response to such stimuli, seasonal exposure may amplify cytokine production in predisposed individuals. These observations suggest that TSLP-driven epithelial activation is context-dependent and may contribute to the variability observed in DAOSD manifestation (11–14).

The absence of significant differences in cytokines such as IL-17A, IL-23, IL-6, IL-2, IFN-γ, TNF-α, and IL-31 is also informative. These mediators primarily reflect downstream inflammatory pathways, many of which are already known to shift during dupilumab therapy. Their lack of discriminatory value in this study suggests that systemic immune alterations alone do not explain ocular susceptibility. Instead, the findings may be consistent with an upstream epithelial contribution, although this cannot be determined from the present data.

Accordingly, TSLP was the only cytokine showing a nominal association with ocular complications, whereas other cytokines did not distinguish between patient groups. This observation supports the hypothesis that ocular complications are not merely a manifestation of global immune imbalance, but are more closely linked to epithelial activation pathways.

Taken together, our findings suggest that stimulated TSLP may represent a potential systemic correlate of ocular complications in dupilumab-treated patients; however, this association did not remain statistically significant after correction for multiple comparisons and should therefore be considered exploratory. Importantly, because all patients with ocular complications had active disease at the time of sampling, the observed differences likely reflect concurrent inflammation rather than a predisposing or predictive factor.

Future studies with larger, multicenter cohorts and integrated assessment of systemic and local ocular biomarkers are required to clarify the role of TSLP in DAOSD development and to determine its potential as a predictive marker.

### Study limitations

Several limitations of this study should be acknowledged.

A major limitation of this study is the heterogeneity of ocular phenotypes. Conjunctivitis, blepharitis, and keratitis were grouped together without standardized grading of severity, ocular surface staining, tear film assessment, or symptom scoring. In addition, detailed ophthalmologic treatment data were not systematically recorded. This heterogeneity limits interpretation of associations between systemic cytokines and ocular disease and represents an important source of potential confounding.

The sample size was relatively small, particularly after stratification according to the presence or absence of ocular complications. Although this is comparable to other exploratory studies on dupilumab-associated ocular surface disease, the limited number of participants reduces statistical power and may have precluded the detection of more subtle differences in cytokine profiles beyond TSLP. Consequently, the findings should be interpreted as hypothesis-generating rather than definitive.

This was a single-center study, which may limit the generalizability of the results to broader or ethnically diverse populations. Environmental exposure patterns, background allergen sensitization, and clinical management of ocular complications may vary across regions, potentially influencing cytokine dynamics.

Plasma cytokine levels may not accurately reflect local ocular surface inflammation, which is predominantly driven by epithelial and tear-film processes. Although plasma cytokine profiling provides valuable insight into systemic immune activation and inducible functional capacity, direct assessment of conjunctival tissue, tear fluid cytokines, or goblet cell markers could offer more precise information on local pathogenic mechanisms underlying dupilumab-associated ocular surface disease.

While cytokines were assessed under both unstimulated and PMA/ionomycin-stimulated conditions, the stimulation protocol is non-specific and does not identify the precise cellular sources of TSLP or other cytokines. Future studies incorporating cell-specific assays or epithelial-focused models may help clarify whether the observed increase in stimulated TSLP reflects epithelial priming, immune cell–derived production, or interactions between both compartments.

Although seasonal sampling was incorporated, environmental exposures were not measured directly. Factors such as pollen counts, humidity, temperature, air pollution, UV exposure, contact lens use, topical eye treatments, and ocular microbiome composition were not systematically evaluated and may have acted as unmeasured confounders, particularly in explaining the stronger association observed during the summer season.

Finally, the cross-sectional comparison between patients with and without ocular complications does not allow conclusions regarding **temporal causality**. It remains unclear whether elevated stimulated TSLP precedes the development of ocular complications or represents a consequence of ocular surface inflammation. Longitudinal studies tracking cytokine profiles before and after the onset of ocular symptoms are needed to establish predictive value. The heterogeneity of ocular diagnoses and the absence of standardized severity grading limit interpretation and may confound associations with systemic cytokines.

Despite these limitations, TSLP represents a hypothesis-generating systemic association that may reflect susceptibility to ocular complications during dupilumab therapy and offers a strong rationale for larger, multicenter, longitudinal investigations integrating systemic and local ocular immune assessments.

These limitations indicate that the present findings should be interpreted as exploratory and hypothesis-generating rather than definitive evidence of a causal or predictive role of TSLP.

## Future direction

Despite these limitations, TSLP represents a hypothesis-generating systemic association that may reflect concurrent systemic immune activation rather than susceptibility to ocular complications during dupilumab therapy and offers a strong rationale for larger, multicenter, longitudinal investigations integrating systemic and local ocular immune assessments.

These limitations indicate that the present findings should be interpreted as exploratory and hypothesis-generating rather than definitive evidence of a causal or predictive role of TSLP.

## Conclusion

Our findings suggest that stimulated TSLP may represent a potential systemic correlate of ocular complications in dupilumab-treated patients; however, this association did not remain significant after correction for multiple comparisons and should therefore be regarded as exploratory. Future studies with larger, multicenter cohorts and integrated analysis of systemic and local ocular biomarkers are required to determine whether TSLP has predictive or mechanistic relevance.

## Data Availability

The raw data supporting the conclusions of this article will be made available by the authors, without undue reservation.
