# Supplementary material for: The evaluation of cytokine profiles in atopic dermatitis patients treated with dupilumab: association with ocular complications
Source: Front Immunol. 2026 Aug 6;17:1873001. doi: 10.3389/fimmu.2026.1873001 (PMC13489922; doi:10.3389/fimmu.2026.1873001)
Supplement: Supplementary file 1 [file DataSheet1.docx]

**SUPPLEMENTARY TABLES**

**Supplementary Table to Table 2.** Due to the non-normal (skewed) distribution of the data, results are presented as median (interquartile range, 25th–75th percentile) for patients with and without eye complications, separately for summer seasons. Values are presented in **pg/mL** under **unstimulated (un)** and **stimulated (s)** conditions.

| **Parameter** | **with ocular complications** | **eye complications no** |
| --- | --- | --- |
| IL4_un | 0 (0–0.1495) | 0 (0–0) |
| IL4_s | 0.018 (0–0.1645) | 0 (0–0) |
| IL13_un | 0 (0–0.174) | 0 (0–0) |
| IL13_s | 0 (0–0.1865) | 0 (0–0.205) |
| IL5_un | 0 (0–0) | 0 (0–0) |
| IL5_s | 0 (0–0.0465) | 0.0255 (0–0.0738) |
| IL10_un | 0 (0–0) | 0 (0–0) |
| IL10_s | 0.178 (0.073–0.466) | 0.3155 (0.1328–0.5548) |
| IL33_un | 0 (0–0) | 0 (0–0) |
| IL33_s | 0 (0–0) | 0 (0–0) |
| IL6_un | 0 (0–3.6795) | 1.752 (0–3.2818) |
| IL6_s | 75.655 (21.23–178.19) | 96.915 (67.35–158.53) |
| IL17_un | 0 (0–5.0615) | 0 (0–0) |
| IL17_s | 377.441 (186.37–681.97) | 278.909 (200.12–398.90) |
| IL12 un | 73.48 (35.32–684.65) | 1.48 (0–49.70) |
| IL12s | 164.85 (36.74–870.24) | 1.48 (0–44.07) |
| TSLP_un | 2.398 (0–16.44) | 0 (0–1.399) |
| TSLP_s | 2.398 (1.265–22.47) | 0.754 (0–1.8875) |
| IL23_un | 0 (0–1138.11) | 0 (0–10.19) |
| IL23_s | 2123.193 (1651.80–2835.02) | 1643.644 (1459.80–1979.41) |
| IL2_un | 0 (0–0.3665) | 0 (0–0) |
| IL2_s | 9905.371 (6505.93–14535.53) | 9445.505 (7644.59–13625.51) |
| IL31_un | 119.602 (0–637.51) | 0 (0–93.86) |
| IL31_s | 127.167 (55.68–581.35) | 95.905 (45.95–155.08) |
| INFgama_un | 1.29 (0–3.4265) | 0 (0–0.9423) |
| INFgama_s | 6548.911 (6051.19–7240.33) | 6874.797 (5989.12–7419.96) |
| TNFalfa_un | 2.51 (0–15.96) | 1.325 (0–3.791) |
| TNFalfa_s | 4054.16 (2297.98–6089.67) | 3677.511 (2694.33–4304.84) |

**Supplementary Table to Table 3.** Due to the non-normal (skewed) distribution of the data, results are presented as median (interquartile range, 25th–75th percentile) for patients with and without eye complications, separately for winter seasons. Values are presented in **pg/mL** under **unstimulated (un)** and **stimulated (s)** conditions.

| **Parameter** | **with ocular complications** | **eye complications no** |
| --- | --- | --- |
| IL4_un | 0 (0–≈0) | 0 (0–0) |
| IL4_s | 0 (0–0.075) | 0.066 (0–0.202) |
| IL13_un | 0 (0–≈0) | 0 (0–0) |
| IL13_s | 0 (0–≈0) | 0 (0–0.212) |
| IL5_un | 0 (0–0) | 0 (0–0) |
| IL5_s | 0 (0–0.145) | 0.079 (0–0.102) |
| IL10_un | 0 (0–0) | 0 (0–0) |
| IL10_s | 0.342 (0.055–0.79) | 0.355 (0.233–0.798) |
| IL33_un | 0 (0–0) | 0 (0–0) |
| IL33_s | 0 (0–0) | 0 (0–0) |
| IL6_un | 0 (0–12.51) | 1.607 (0–4.69) |
| IL6_s | 192.549 (100.25–252.40) | 163.18 (103.35–333.98) |
| IL17_un | 0 (0–31.10) | 0 (0–0) |
| IL17_s | 434.106 (226.44–681.54) | 276.1 (211.33–541.43) |
| IL12 un | 128.76 (0–462.46) | 0 (0–155.10) |
| IL12s | 164.863 (29.07–501.92) | 28.28 (0–164.85) |
| TSLP_un | 3.94 (0–11.08) | 0 (0–3.7) |
| TSLP_s | 4.348 (1.72–13.34) | 1.501 (0–3.93) |
| IL23_un | 0 (0–203.98) | 0 (0–49.62) |
| IL23_s | 2041.008 (1490.79–2584.25) | 2065.771 (1716.18–2474.54) |
| IL2_un | 0 (0–0) | 0 (0–0) |
| IL2_s | 16046.95 (9602.99–16471.82) | 15270.85 (13229.43–24317.53) |
| IL31_un | 182.899 (0–333.93) | 0 (0–94.88) |
| IL31_s | 215.414 (51.21–358.60) | 99.973 (40.61–159.65) |
| INFgama_un | 0 (0–2.7) | 0 (0–0.618) |
| INFgama_s | 7380.069 (7062.79–7660.77) | 7432.178 (7281.60–8768.62) |
| TNFalfa_un | 2.705 (0–9.81) | 3.706 (0–6.96) |
| TNFalfa_s | 4929.205 (4088.15–7277.40) | 5248.557 (4109.96–7688.61) |

**Supplementary Table to Table 5 – summer season**

ROC analysis is performed with the TSLP for the summer period – with the setting that higher values indicate eye damage. We show the empirical AUC estimate: AUC=0.806, 95% Confidence limit: (0.467, 0.938), significance p=0.0025 (test AUC ≤ 0.5 vs alternative AUC > 0.5**).** ROC analysis for the summer dataset included a subset of patients (n = 19) due to missing data for stimulated TSLP measurements in some samples.

**Area Under Curve Analysis (Empirical Estimation)**

Estimated Prevalence = 9 / 19 = 0.4737

**Upper One-Sided Test of	95% Confidence**

**H0: AUC ≤ 0.5 vs. H1: AUC > 0.5	Interval Limits**

**Standard	──────────────────────	────────────**

**Criterion	Count	AUC	Error	Z-Value	P-Value	Lower	Upper**

──────────────────────────────────────────────────────────────────────────────────────────────────────────────────

TSLP_s	19	0.8056	0.1089	2.806	0.0025	0.467	0.9381

**The most appropriate cutoff value if TSLP were to be used as a diagnostic test (values ≥ 1.21 or ≥ 1.32).**

**Common Rates and Indices for Each Cutoff Value**

**Table Counts**

**───────────────────**

**Cutoff	TPs	FPs	FNs	TNs	TPR	TNR	TPR +**

**Value	A	B	C	D	(Sensitivity)	(Specificity)	PPV	Accuracy	TNR**

≥ 1.21	8	3	1	7	0.8889	0.7	0.7273	0.7895	1.5889

≥ 1.32	7	2	2	8	0.7778	0.8	0.7778	0.7895	1.5778

**Counts, Proportion Correctly Classified, Proportion Incorrectly Classified, Estimated Prevalence**

**Table Counts**

**───────────────────	Proportion Classified**

**Cutoff	TPs	FPs	FNs	TNs	─────────────────**

**Value	A	B	C	D	Correctly	Incorrectly**

──────────────────────────────────────────────────────────────────────────────

≥ 1.21	8	3	1	7	0.7895	0.2105

≥ 1.32	7	2	2	8	0.7895	0.2105

Definitions:

Cutoff Value	Indicates the criterion value range that predicts a positive condition.

A	The number of True Positives.

B	The number of False Positives.

C	The number of False Negatives.

D	The number of True Negatives.

Proportion Correctly Classified	Accuracy = (A + D) / (A + B + C + D).

Proportion Incorrectly Classified	Inaccuracy = (B + C) / (A + B + C + D).

**ROC Plot** **TSLP – Eye complications, summer season**

**Supplementary Table to Table 5 – winter season**

**ROC analysis is performed with the TSLP for the winter season. We show the empirical AUC estimate: AUC=0.7, 95% Confidence limit: (0.416, 0.86), significance p=0.0353.**

**Area Under Curve Analysis (Empirical Estimation)**

Estimated Prevalence = 11 / 26 = 0.4231

**Upper One-Sided Test of	95% Confidence**

**H0: AUC ≤ 0.5 vs. H1: AUC > 0.5	Interval Limits**

**Standard	──────────────────────	────────────**

**Criterion	Count	AUC	Error	Z-Value	P-Value	Lower	Upper**

────────────────────────────────────────────────────────────────────────────────────────────────────────────────

TSLP_s	26	0.7	0.1106	1.808	0.0353	0.4155	0.8598

**The most appropriate cutoff value if TSLP s were to be used as a diagnostic test (values ≥ 3.94).**

**Common Rates and Indices for Each Cutoff Value**

**Table Counts**

**───────────────────**

**Cutoff	TPs	FPs	FNs	TNs	TPR	TNR	TPR +**

**Value	A	B	C	D	(Sensitivity)	(Specificity)	PPV	Accuracy	TNR**

≥ 3.94	7	3	4	12	0.6364	0.8000	0.7000	0.7308	1.4364

**Counts, Proportion Correctly Classified, Proportion Incorrectly Classified, Estimated Prevalence**

**Proportion Classified**

**Cutoff	TPs	FPs	FNs	TNs	─────────────────**

**Value	A	B	C	D	Correctly	Incorrectly**

──────────────────────────────────────────────────────────────────────────────

≥ 3.94	7	3	4	12	0.7308	0.2692

Definitions:

Cutoff Value	Indicates the criterion value range that predicts a positive condition.

A	The number of True Positives.

B	The number of False Positives.

C	The number of False Negatives.

D	The number of True Negatives.

Proportion Correctly Classified	Accuracy = (A + D) / (A + B + C + D).

Proportion Incorrectly Classified	Inaccuracy = (B + C) / (A + B + C + D).

**ROC plot for TSLP – winter season**
